# ICT-based system to predict and prevent falls (iStoppFalls): results from an international multicenter randomized controlled trial

**DOI:** 10.1186/s11556-015-0155-6

**Published:** 2015-11-27

**Authors:** Yves J. Gschwind, Sabine Eichberg, Andreas Ejupi, Helios de Rosario, Michael Kroll, Hannah R. Marston, Mario Drobics, Janneke Annegarn, Rainer Wieching, Stephen R. Lord, Konstantin Aal, Daryoush Vaziri, Ashley Woodbury, Dennis Fink, Kim Delbaere

**Affiliations:** Neuroscience Research Australia, University of New South Wales, Barker Street, Randwick, Sydney, New South Wales 2031 Australia; Institute of Movement and Sport Gerontology, German Sport University Cologne, Am Sportpark Muengersdorf 6, 50933 Cologne, Germany; Assistive Healthcare Information Technology Group, Austrian Institute of Technology, Donau-City-Strasse 1, 1220 Vienna, Austria; Institute of Biomechanics of Valencia, University Polytechnic of Valencia, Edificio 9C Camino de Vera s/n, 46022 Valencia, Spain; Biomedical Research Networking Center in Bioengineering, Biomaterials and Nanomedicine (CIBER-BBN), Healthcare Technology Group, Valencia, Spain; Personal Health Department, Philips Research Europe, High Tech Campus 34, 5656AE Eindhoven, The Netherlands; Institute for Information Systems, University of Siegen, Hölderlinstrasse 3, 57076 Siegen, Germany

**Keywords:** Fall prevention, Older adults, Exergames, Strength, Balance, Sensor-based

## Abstract

**Background:**

Falls and fall-related injuries are a serious public health issue. Exercise programs can effectively reduce fall risk in older people. The iStoppFalls project developed an Information and Communication Technology-based system to deliver an unsupervised exercise program in older people’s homes. The primary aims of the iStoppFalls randomized controlled trial were to assess the feasibility (exercise adherence, acceptability and safety) of the intervention program and its effectiveness on common fall risk factors.

**Methods:**

A total of 153 community-dwelling people aged 65+ years took part in this international, multicentre, randomized controlled trial. Intervention group participants conducted the exercise program for 16 weeks, with a recommended duration of 120 min/week for balance exergames and 60 min/week for strength exercises. All intervention and control participants received educational material including advice on a healthy lifestyle and fall prevention. Assessments included physical and cognitive tests, and questionnaires for health, fear of falling, number of falls, quality of life and psychosocial outcomes.

**Results:**

The median total exercise duration was 11.7 h (IQR = 22.0) over the 16-week intervention period. There were no adverse events. Physiological fall risk (Physiological Profile Assessment, PPA) reduced significantly more in the intervention group compared to the control group (F_1,127_ = 4.54, *p* = 0.035). There was a significant three-way interaction for fall risk assessed by the PPA between the high-adherence (>90 min/week; *n* = 18, 25.4 %), low-adherence (<90 min/week; *n* = 53, 74.6 %) and control group (F_2,125_ = 3.12, *n* = 75, *p* = 0.044). Post hoc analysis revealed a significantly larger effect in favour of the high-adherence group compared to the control group for fall risk (*p* = 0.031), postural sway (*p* = 0.046), stepping reaction time (*p* = 0.041), executive functioning (*p* = 0.044), and quality of life (p for trend = 0.052).

**Conclusions:**

The iStoppFalls exercise program reduced physiological fall risk in the study sample. Additional subgroup analyses revealed that intervention participants with better adherence also improved in postural sway, stepping reaction, and executive function.

**Trial registration:**

Australian New Zealand Clinical Trials Registry Trial ID: ACTRN12614000096651

International Standard Randomised Controlled Trial Number: ISRCTN15932647

## Background

Falls and the often costly treatment of fall-related injuries are a burden for older people’s autonomy and independence as well as the healthcare system [1]. Previous reviews and meta-analyses have shown that a range of successful strategies for fall prevention is available [2, 3]. In order to prevent falls in older people, it is important to implement effective preventative programs on a broad scale.

It is well known from the literature that exercise interventions can reduce the risk of falling and rate of falls provided they are continued over a longer period of at least six months [2, 4]. However, incorporating a new exercise regimen into daily life can be challenging for many older people due to poor exercise tolerance and enjoyment. Videogame technologies provide an opportunity to deliver exercise programs (exergaming) by offering increased convenience and greater level of engagement. Furthermore, several studies have suggested that their effectiveness towards improving key fall risk factors may be equivalent to traditional exercise programs of similar content and dosage [5–7]. A recent systematic review provided preliminary evidence for the effectiveness of such innovative exercise modes on physical and cognitive factors associated with fall risk in older people [8]. Furthermore, sensor-based virtual environments allow inclusion of dual-tasking to support task-specific training (e.g., cognitive tasks), continuous movement monitoring and real-time performance feedback which may improve long-term exercise adherence in older people [9]. However, there is a lack of evidence on how fall prevention can be successfully implemented into the community, especially in older people who would like to exercise at home on their own [4].

Despite promising evidence regarding the use of virtual reality and ambient-assistive technologies to deliver exercise programs in people’s homes, off-the-shelf exergame systems such as the Nintendo Wii or Microsoft Xbox are not sufficiently tailored to the specific circumstances and values of older people. Therefore, there is a need to design customized programs [10]. We developed a new Information and Communication (ICT)-based system for fall risk assessment and fall prevention in older people living independently at home, called iStoppFalls (www.istoppfalls.eu). The present study investigated the feasibility and effectiveness of the individually tailored ICT-based iStoppFalls exergame program delivered through the home television (TV) on fall risk factors. We hypothesized that this newly developed ICT-based system for fall prevention at home is feasible for older people in terms of exercise adherence, acceptability and safety. We also hypothesised that regular use of the iStoppFalls exercise program would lead to improved balance and strength outcomes (physiological fall risk) and quality of life. In addition, the exercise training may also be effective on the improvement of cognitive fall risk factors in older people.

## Methods

### Study design

**Fig. 1:**
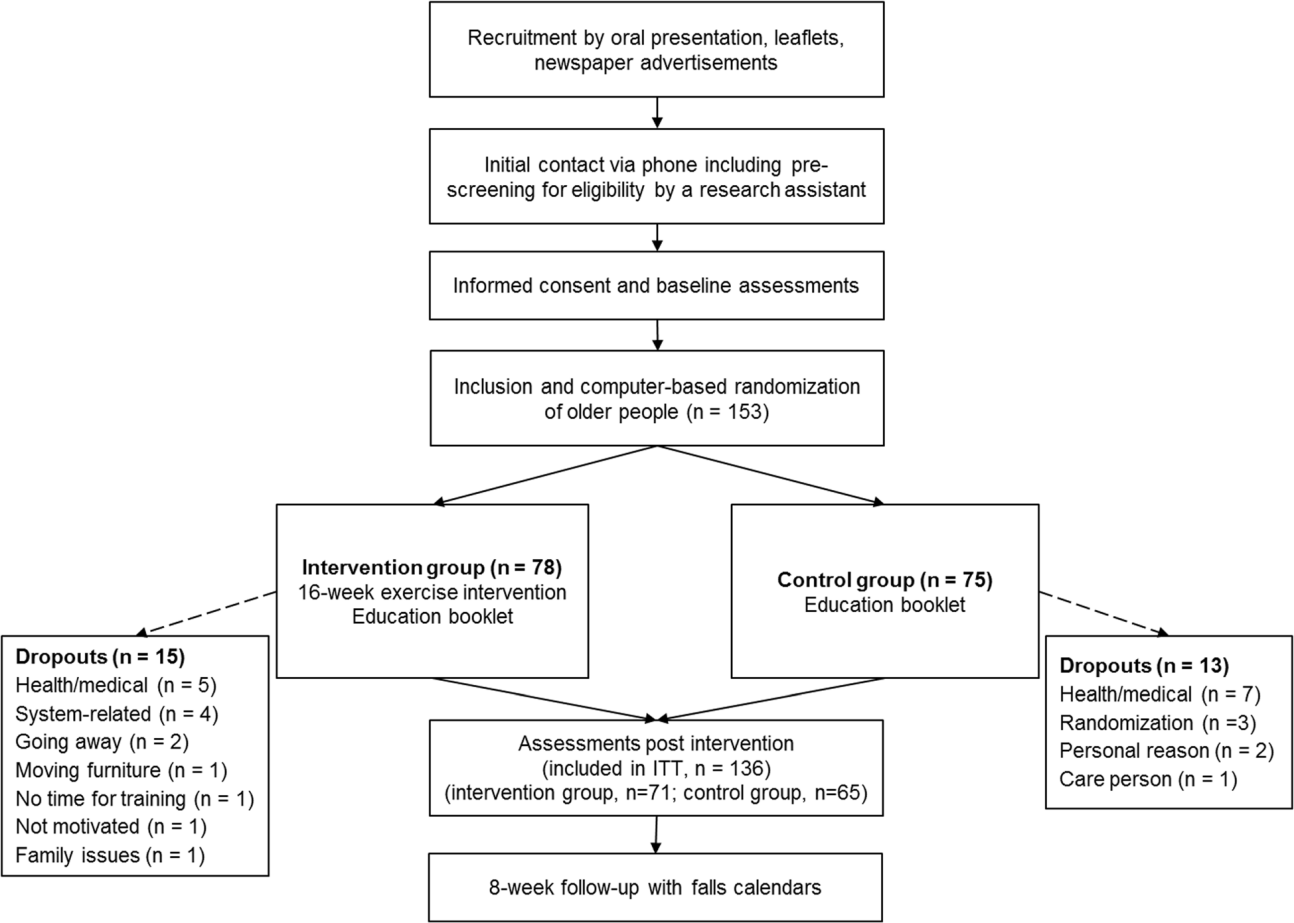
Study flow chart

One hundred fifty-three community-dwelling older people aged 65 years and older took part in this international, multicentre, single-blinded, two-group randomized trial (Fig. 1). Study sites were located in Germany (Cologne), Spain (Valencia) and Australia (Sydney). The trial was conducted between January and October 2014. A study protocol describing the applied system and methodologies in more detail is available elsewhere [11].

### Participants

Older people were included if they met the following eligibility criteria: (1) aged 65 years and older, (2) living in the community, (3) able to walk 20 m without a walking aid, (4) able to watch television TV with or without their glasses from 3 m distance, and (5) have enough space for system use (3.5 m^2^). The exclusion criteria were: (1) insufficient language skills to understand the study procedures, (2) cognitive impairment (Mini-Cog: 1–2 recalled words and abnormal clock drawing test) [12], and (3) medical conditions precluding participation in a regular exercise program (i.e., uncontrolled hypertension, severe neurological disorder, acute cancer, psychiatric disorder, acute infection).

### Randomization and blinding

Following baseline assessments, eligible participants were randomised by permuted block-randomisation (ratio 1:1) using a unique computer-generated random number for identification. Participants who lived in the same household were treated as one unit and randomised into the same block. Research staff performing the assessments was experienced, trained and blinded to group allocation. Participants were reminded not to talk about their user experience to avoid unblinding.

### Protocol

All participants gave written informed consent prior to inclusion. Ethical approval was obtained by the ethics committees of the German Sport University Cologne (24.09.2013), the Polytechnic University of Valencia (19.12.2013), and the Human Research Ethics Committee of the University of New South Wales (reference number HC12316, 19.12.2013). After baseline assessment (see outcome measures described below), participants were formally entered into the study and randomised to intervention or control groups (Fig. 1). Participants in both groups received an evidence-based educational booklet about general health and fall prevention [13]. Control group participants did not receive any additional intervention and were encouraged to follow their habitual exercise routines if applicable. Participants in the intervention group were instructed in the use of the iStoppFalls program in their home, including tailored and targeted (personalised) balance and muscle strength exercises (exergames), and a test battery for the assessment of individual fall risk. Trained research staff installed the iStoppFalls system components including a personal computer (Shuttle Barebone Slim-PC), a Google TV set top box (STB) by Sony, a Microsoft Kinect (3D depth sensor), a Senior Mobility Monitor (SMM) by Philips (3D accelerometer, barometer) [14], and a Nexus 7 Android tablet in the homes of intervention group participants. Participants were instructed how to control the movements of a virtual avatar on the TV screen and how to navigate through the system with gestures, voice control or use of the tablet. Two weeks after system installation, a second home visit ensured correct and safe system use as well as progression of training. Phone support was available throughout the 16-week intervention and additional home visits were offered if required.

### Exergames

Intervention group participants conducted a 16-week exercise program based on best practice recommendations for exercise to prevent falls in older people by Sherrington et al. [15, 16]. The recommended training dose consisted of at least three balance sessions of about 40 min each (including each of the exergames) and at least three muscle strength sessions of about 15 to 20 min each (including all strength exercises) per week. A sheet of ‘Exercise Safety Guidelines’ was given to each participant by an experienced researcher. Adherence was monitored automatically by the iStoppFalls system. Participants received individual training and assessment reminders through the tablet computer and by an unblinded research assistant if required. All aggregated data were transmitted to a knowledge-based system (server) which allowed participants to continuously monitor their fall risk and results via the STB and tablet computer at home.

Balance exercises were based on the Weight-bearing Exercise for Better Balance (WEBB) program (www.webb.org.au). Three balance exergames ‘Bumble Bee Park’, ‘Hills & Skills’, and ‘Balance Bistro’ for walking, weight shifting, knee bending, and/or stepping in different directions were specifically developed for the iStoppFalls project. Additionally, cognitive tasks targeting semantic and working memory (e.g., remembering objects) were added once a participant reached higher exergame levels (dual-tasking). Progression was achieved by reducing upper limb support, narrowing the base of support, adjusting speed of movement, increasing gaming duration, and proceeding to a higher difficulty level.

Strength exercises for the lower extremities including knee extension, knee flexion, hip abduction, calf raises, and toe raises were based on the strength exercise component of the Otago exercise program [17]. Between 2 and 3 sets of 10 to 15 repetitions and rest periods of 1 min were recommended. Progression was achieved by increasing the number of repetitions, the number of sets, and the difficulty level (e.g., by using 1–3 kg ankle cuff weights).

### Outcome

The whole sample was assessed at baseline (0 weeks) and at the end of the intervention period (16 weeks). A self-report questionnaire was used to collect information on socio-demographic characteristics and medical history. Anthropometrics were assessed as part of the baseline assessments. Falls frequency and adverse events were monitored with monthly diaries for 6 months. Participants were contacted by phone when the diaries were not returned.

### Primary outcome measures

The short version of the Physiological Profile Assessment (PPA) estimated individual fall risk based on five sensorimotor tests: contrast sensitivity (Melbourne edge test (MET), peripheral sensation (proprioception), balance (sway when standing on medium-density foam with eyes open), lower extremity muscle strength (knee extension), and hand reaction time (HRT) [18]. The European Quality of Life 5 Dimensions (EQ-5D) questionnaire was used to assess health status in five dimensions: mobility, self-care, usual activities, pain/discomfort, and anxiety/depression (www.euroqol.org/eq-5d-products/eq-5d-5l.html).

### Secondary outcome measures

#### Health measures

The 12-item World Health Organization Disability Assessment Schedule (WHODAS) 2.0 was used to assess general health (understanding and communicating, mobility, self-care, interpersonal interactions, household and work activities, and participation in society) [19]. The 9-item Patient Health Questionnaire (PHQ-9) was used to assess the severity of depression [20]. Participants’ concerns about falling for 10 daily activities were investigated by the shortened Iconographical Falls Efficacy Scale (Icon-FES) [21]. The Incidental and Planned Activity Questionnaire (IPEQ) in Spain and Australia [22], and the Physical Activity Questionnaire for the population aged 50 years and older (PAQ-50+) in Germany [23] were applied to retrospectively assess physical activity patterns. The SMM was used in the intervention group participants to detect walking distance and sit-to-stand transfers during daily life activities, using previously defined algorithms [24]. Participants were asked to wear the SMM during waking hours. The generated peak power was calculated for each detected sit-to-stand transfer, with an expected detection sensitivity of 89 % [24].

#### Physical measures

In addition to the PPA, tests for coordinated stability and maximal balance range were assessed as measures of dynamic balance. Tests for static balance (e.g., tandem stance), walking speed over 4 m, and five times chair stand performance from the Short Physical Performance Battery (SPPB) [25] were also administered. The timed up and go test (TUG) was used as a combination of basic functionality, physical mobility, and dynamic balance [26–28]. Steady-state walking speed was measured over a 10 m distance (plus 2 m for acceleration and 2 m for deceleration) with a stop watch [29]. Dual-tasking ability was assessed by asking participants to count backwards by three starting from a random 3-digit number while walking over a 10 m distance [30]. Furthermore, four specifically developed sensor-based physical tests were performed: 1) balance (bipedal, semi-tandem, near-tandem, and tandem stance); 2) arm reaction time (hitting two randomly flashing lights on a virtual table by lifting one arm), 3) stepping reaction time (step on two randomly flashing lights on a virtual floor); and 4) five times sit-to-stand (stand up and sit down).

#### Cognitive measures

The Trail Making Test (TMT) was performed as a measure of executive function (divided attention), processing/motor speed, and mental flexibility, in which participants had to connect numbers and lines in order [31, 32]. Cognitive control was assessed by a computer-based Victoria Stroop Test (VST) using the Psychology Experiment Building Language (PEBL) software version 0.13 (http://pebl.sourceforge.net/). In the VST, participants had to maintain a goal in mind and supress habitual responses to correctly identify coloured dots and words [32]. The Digit Symbol Coding Test (DSC) required participants to copy symbols to investigate processing speed [33]. Working memory, attention, and concentration was measured by the Digit Span Backward (DSB) [33]. In this test, participants had to repeat numbers in the reverse order [32]. A computer-based Attention Network Test (ANT) where participants had to determine whether a central arrow points to the left or right was used on PEBL to quantify the processing efficiency within three attentional networks: alerting, orienting and executive attention [34].

#### Technology use measures

Usability and enjoyment was assessed by the 10-item System Usability Scale (SUS) [35] and the 8-item Physical Activity Enjoyment Scale (PACES) [36, 37]. A score of 0–24 on the PACES corresponds to a lower overall enjoyment, and a score of 24–48 corresponds to a higher enjoyment. Overall usability measured by the SUS ranges from 0 “worst imaginable” to 100 “best imaginable”. The Dynamic Acceptance Model for the Re-evaluation of Technologies (DART) was used for the analysis and evaluation of user acceptance of products or services [38]. It comprises items related to appeal, consistency, operation, speed, language and usability on a 6-point Likert scale (6 indicates “very important”/”totally fulfilled”).

### Statistical analyses and sample size

Sample size calculations based on PPA as the primary outcome estimated a sample size of 52 participants (f = 0.40, alpha 5 %, power 80 %) [39]. With an anticipated dropout rate of 15 %, the recruitment aimed for 60 participants per site. For this study the intention-to-treat (ITT) method including post-assessment data from exercise dropouts was applied. Data on feasibility was analysed using descriptive techniques. Student t-tests for continuous variables with normal distribution, chi-square test for nominal data, and Mann–Whitney U test for ordinal or continuous data without normal distribution were used to determine differences between the intervention and control group at baseline. Repeated measures ANOVA was used to determine the intervention effect on outcome measures at follow-up. Three-way (comparing 3 groups) and two-way (comparing high-adherence group to control group) repeated mesuares ANOVAs were used to perform subgroup analyses for grouping variables of interest (exercise adherence and fall risk based on median split). The two-sided alpha level was 5 %. Analyses were performed with SPSS version 23 for Windows (SPSS, Inc., Chicago, IL).

## Results

The flow of participants through the trial is illustrated in Fig. 1. One hundred fifty three older people were included in this study (*n* = 78 intervention group, *n* = 75 control group); 136 participants were reassessed after 16 weeks (*n* = 28 exercise dropouts, of which *n* = 11 agreed to be reassessed so that their data could be included in the ITT analyses). Baseline characteristics of the sample, showing no marked differences between groups, are displayed in Table 1. For the six month study period, a total of 24 falls were reported by monthly fall calendars, with eight falls being reported in the intervention group and 15 falls in the control group. No falls were related to using the iStoppFalls system. No other adverse events were reported.

**Table 1:** Participants’ characteristics at baseline presented as Mean ± Standard Deviation (SD) or number (percentage)

Characteristic	Overall (*n* = 153)	Intervention group (*n* = 78)	Control group (*n* = 75)
Age, years	74.7 ± 6.3	74.7 ± 6.7	74.7 ± 6.0
Female, n (%)	93 (61.2 %)	43 (55.8 %)	50 (66.7 %)
Body mass index, kg/m^2^	26.3 ± 3.8	26.1 ± 3.8	26.5 ± 3.9
Education, years	11 ± 5	12 ± 5	11 ± 4
Medication use (n)	3.3 ± 2.8	3.5 ± 3.1	3.1 ± 2.5
One or more falls in the previous year, n (%)	51 (34 %)	25 (32.5 %)	26 (35.6 %)
Comorbidities, number	3.0 ± 1.4	2.9 ± 1.3	3.1 ± 1.5
Heart problems, n (%)	21 (14.1 %)	12 (15.6 %)	9 (12.5 %)
High blood pressure, n (%)	78 (53.1 %)	35 (46.1 %)	43 (60.6 %)
Osteoporosis, n (%)	35 (23.3 %)	18 (23.4 %)	17 (23.3 %)
Lower back pain, n (%)	61 (40.9 %)	33 (43.4 %)	28 (38.4 %)
Hip pain, n (%)	33 (22.4 %)	16 (21.1 %)	17 (23.9 %)
Knee and/or leg pain, n (%)	62 (41.6 %)	29 (37.7 %)	33 (45.8 %)
Foot pain, n (%)	36 (24.0 %)	18 (23.4 %)	18 (24.7 %)
PAQ-50+, hours	41.62 ± 21.20	40.55 ± 23.62	42.80 ± 18.70
IPEQ, hours	26.45 ± 18.55	29.56 ± 16.91	23.13 ± 19.80
WHODAS, score	15.79 ± 4.31	16.00 ± 4.52	15.57 ± 4.10
Computer ownership, n	111 (72.6 %)	58 (74.4 %)	53 (70.7 %)

*IPEQ* Incidental and Planned Activity Questionnaire, *PAQ-50+* Physical Activity Questionnaire for the population aged 50 years and older, *VAS* Visual Analogue Scale, *WHODAS* World Health Organization Disability Assessment Schedule

### Adherence and user experience

Over the 16-week training period, participants in the intervention group used the iStoppFalls system 42 times (median, interquartile range IQR = 57) at a game level of 2.1 (median, IQR = 3.9) for a total duration of 11.7 h (median, IQR = 22.0) including and 7.0 h (median, IQR = 12.8) excluding instructions, respectively. Eighteen participants exercised for a total of 24 h over the 16-week training period (1.5 h per week), of which two participants exceeded the recommended training dose of 48 h (3 h per week) and another four participants exercised for over 40 h (2.5 h per week). Of the remaining intervention group participants, 12 exercised for at least 16 h in total (1 h per week), 18 participants exercised for at least 8 h in total (30 min per week) and 30 participants exercised for less than 8 h in total. Balance exergames were performed 24 times (median, IQR = 30) at an average game level of 2.6 (median, IQR = 8.0) for a total duration of 4.0 h (median, IQR = 6.9) including instructions and 2.8 h (median, IQR = 4.1) excluding instructions. Strength exercises were performed 20 × (median, IQR = 31) at an average intensity level of 1.4 (median, IQR = 2.0) for a duration of 7.9 h (median, IQR = 13.4) including instructions and 4.4 h (median, IQR = 7.7) excluding instructions. Outcomes from the SMM were based on data analysis of 63 devices. Total SMM wearing time was 580 ± 459 h with a walking distance of 145 ± 45 m per hour and a generated peak power of 0.28 ± 0.07 arbitrary units across an average of 1.6 ± 1.4 detected sit-to-stand transfers per hour.

A mean score of 31 (standard deviation SD = 8) on the PACES suggested higher levels of enjoyment and a mean score of 62 (SD = 23) on the SUS suggested acceptable usability. Results for the DART questionnaire showed that the exergames were the most highly rated in terms of appeal, consistency, operation, speed, language and usability with a score of 4.4 points.

### Effectiveness of the intervention

#### Intervention and control group comparison

Table 2 displays between-group differences after 16 weeks of intervention. Fall risk assessed by the PPA was significantly reduced in the intervention group compared with the control group (F_1,127_ = 4.54, *p* = 0.035). Hand reaction time worsened significantly in the control group compared to the intervention group (F_1,128_ = 9.59, *p* = 0.002). The reduction in time for 10 m walking while counting backwards by three was more pronounced in the intervention than the control group (F_1,127_ = 4.32, *p* = 0.040).

**Table 2:** Group comparison between baseline and post-assessment (Mean ± Standard Deviation, SD)

Variable	Intervention group (*n* = 78)		Control group (*n* = 75)		
	Pre	Post	Pre	Post	*P* ^a^
Primary outcome measures					
Physiological Profile Assessment (score)	0.62 ± 0.89	0.41 ± 0.95	0.55 ± 0.90	0.39 ± 0.80	0.035
European Quality of Life – 5 Dimensions (index)	0.86 ± 0.11	0.86 ± 0.15	0.86 ± 0.13	0.87 ± 0.13	0.741
European Quality of Life – 5 Dimensions (VAS)	79.2 ± 14.7	80.9 ± 13.7	81.7 ± 12.7	79.9 ± 14.6	0.244
Secondary outcome measures					
General health measures					
WHODAS (score)^b^	16.0 ± 4.5	16.3 ± 4.8	15.6 ± 4.1	16.0 ± 4.5	0.494
Physical and sensorimotor measures					
Melbourne edge test (score)	20.8 ± 1.6	21.0 ± 2.1	21.1 ± 1.8	21.2 ± 2.1	0.278
Proprioception (°)^b^	1.3 ± 1.0	1.2 ± 1.1	1.7 ± 1.3	1.3 ± 0.9	0.758
Knee extension strength (kg)	23.0 ± 9.2	28.0 ± 11.9	22.7 ± 9.9	27.1 ± 14.0	0.545
Hand grip strength (kg)	24.9 ± 9.4	23.8 ± 8.6	23.9 ± 9.0	24.0 ± 8.7	0.979
Hand reaction time (ms)	245 ± 43	249 ± 47	239 ± 50	263 ± 45	0.002
Sway, area (mm^2^)^b^	841 ± 692	774 ± 1191	797 ± 596	629 ± 973	0.940
Coordinated stability, errors^b^	11.3 ± 10.3	8.5 ± 9.0	12.2 ± 13.6	8.0 ± 9.0	0.909
Maximum balance range, anterior-posterior (mm)	139 ± 41	145 ± 35	137 ± 48	152 ± 39	0.713
Timed up and go test (s)	9.7 ± 2.8	9.5 ± 2.7	10.2 ± 3.1	9.2 ± 2.1	0.504
Short Physical Performance Battery (score)	10.4 ± 1.7	11.1 ± 1.5	10.2 ± 1.6	10.9 ± 1.4	0.548
Sensor-based chair stand test (s)^b^	12.5 ± 4.2	10.8 ± 3.2	13.1 ± 3.7	12.1 ± 3.3	0.347
Sensor-based semi tandem stance (s)	28.3 ± 6.0	28.9 ± 5.2	29.5 ± 3.0	29.2 ± 4.5	0.412
Sensor-based near tandem stance (s)	28.1 ± 6.3	28.5 ± 5.7	28.3 ± 5.4	29.1 ± 4.5	0.876
Sensor-based full tandem stance (s)	24.4 ± 9.9	24.4 ± 9.5	23.3 ± 10.2	25.0 ± 9.2	0.727
Sensor-based hand reaction time (ms)^b^	698 ± 142	619 ± 131	705 ± 391	649 ± 162	0.259
Sensor-based stepping reaction time (ms)^b^	794 ± 199	732 ± 209	801 ± 142	743 ± 121	0.055
10 m walking time, single-tasking (s)^b^	8.9 ± 2.0	8.7 ± 1.8	8.9 ± 2.3	8.6 ± 1.8	0.323
10 m walking time, dual-tasking (s)^b^	12.0 ± 3.9	11.4 ± 3.8	12.0 ± 4.5	11.7 ± 4.0	0.040
Cognitive measures					
Trail Making Test, part A (s)^b^	42.8 ± 17.5	40.4 ± 16.5	44.2 ± 18.1	39.5 ± 14.1	0.736
Trail Making Test, part B (s)^b^	114.5 ± 55.8	100.6 ± 51.1	116.2 ± 52.2	96.3 ± 47.9	0.474
Digit symbol coding test, correct (n)	49.4 ± 15.6	51.4 ± 15.7	44.5 ± 13.0	48.6 ± 11.9	0.444
Digit span backward test, score (n)	6.0 ± 2.3	6.1 ± 2.1	5.9 ± 2.3	5.7 ± 2.5	0.178
Attention Network Test, reaction time (ms)^b^	808 ± 134	789 ± 130	810 ± 116	745 ± 97	0.121
Attention Network Test, alert (ms)	29.5 ± 34.3	37.3 ± 30.0	35.4 ± 33.8	39.1 ± 32.2	0.262
Attention Network Test, orient (ms)	44.4 ± 38.2	44.0 ± 40.1	51.5 ± 42.1	59.0 ± 40.6	0.561
Attention Network Test, conflict (ms)^b^	134.3 ± 74.7	117.3 ± 53.3	127.1 ± 60.3	126.7 ± 63.5	0.329
Victoria Stroop Test, intrusions (n)^b^	3.0 ± 3.4	2.3 ± 3.4	3.2 ± 4.0	2.6 ± 4.3	0.802
Victoria Stroop Test, efficacy (colour words/words)^b^	1.69 ± 0.73	1.52 ± 0.67	1.63 ± 0.74	1.48 ± 0.60	0.412
Psychological measures					
Iconographical – Falls Efficacy Scale (score)	16.2 ± 5.3	12.6 ± 8.5	15.4 ± 4.4	11.0 ± 7.5	0.593
Patient Health Questionnaire – 9 (score)^b^	3.06 ± 3.14	2.48 ± 2.92	2.99 ± 2.97	2.58 ± 2.38	0.340

*VAS* Visual Analogue Scale, *WHODAS* World Health Organization Disability Assessment Schedule

^a^ANOVA (group*time effect), ^b^Log transformed

Higher scores are better for European Quality of Life – 5 Dimensions (index and VAS), WHODAS, Melbourne edge test, knee extension strength, hand grip strength, maximum balance range, all sensor-based tandem stances, digit symbol coding test and digit span backward test (for all other scores lower values indicate performance improvement)

#### Pre-planned subgroup analyses

Tables 3 and 4 shows the results of the pre-planned subgroup analyses for exercise dose. There was a significant three-way interaction for fall risk assessed by the PPA between the high-adherence (>90 min exercise per week), low-adherence (<90 min exercise per week) and control group (F_2,125_ = 3.12, *p* = 0.044). Two-way analyses comparing the high-adherence group with the control group revealed a significant larger effect in favour of the high-adherence group for fall risk (F_1,77_ = 4.82, *p* = 0.031), postural sway (F_1,75_ = 4.13, *p* = 0.046), executive function (F_1,71_ = 4.21, *p* = 0.044) as well as sensor-based full tandem stance time (F_1,73_ = 4.35, *p* = 0.040) and stepping reaction time (F_1,75_ = 4.40, *p* = 0.041). There was also a trend for an improvement in quality of life (F_1,73_ = 3.91, *p* = 0.052, borderline) when the high-adherence group was compared to the control group.

**Table 3:** Participant characteristics at baseline (mean ± Standard Deviation, SD) comparing subgroups of low and high adherence

	High-adherence >90 min (*n* = 18)	Low-adherence <90 min (*n* = 53)	Baseline comparison
			*p*
Age, years	73.1 ± 6.7	74.9 ± 6.3	0.556
Female, n (%)	12 (66.7 %)	26 (49.1 %)	0.112
Body mass index, kg/m2	24.8 ± 3.5	26.5 ± 4.0	0.223
Education, years	12 ± 5	11 ± 5	0.906
Medication use (n)	2.6 ± 2.4	3.6 ± 3.1	0.368
One or more falls in the previous 12 months, n (%)	6 (33.3 %)	16 (30.8 %)	0.852
Comorbidities, number	2.8 ± 1.2	2.9 ± 1.4	0.747
WHODAS, score	14.9 ± 3.0	15.7 ± 4.1	0.746

WHODAS World Health Organization Disability Assessment Schedule

**Table 4:** Subgroup analyses for exercise adherence Group comparison between baseline and post-assessment (Mean ± Standard Deviation)

	High-adherence >90 min (*n* = 18)		Low-adherence <90 min (*n* = 53)		Baseline comparison	Pre-post comparison	
	Pre	Post	Pre	Post		3-way	2-way^a^
Primary outcome measures					*p*	*p*	*p*
Physiological Profile Assessment, score	0.84 ± .85	0.39 ± .77	0.55 ± 0.93	0.34 ± .91	0.430	0.044	0.031
European Quality of Life – 5 Dimensions, VAS	70.7 ± 13.9	80.3 ± 13.17	80.6 ± 14.6	80.8 ± 14.18	0.057	0.099	0.052
Selected secondary outcome measures							
Sway area, mm^2^	1093 ± 778	554 ± 487	754.9 ± 662	718 ± 1048	0.150	0.057	0.046
Sensor-based full tandem stance, s	25.0 ± 10.0	26.5 ± 8.0	25.2 ± 9.5	23.1 ± 10.0	0.568	0.092	0.040
Knee extension strength, kg	20.1 ± 6.4	27.8 ± 15.8	24.5 ± 9.8	28.0 ± 10.6	0.277	0.425	0.291
Sensor-based chair stand test, s	11.1 ± 2.9	10.4 ± 3.0	12.6 ± 4.4	10.8 ± 10.8	0.133	0.053	0.983
Sensor-based stepping reaction time, ms	756 ± 161	657 ± 107	788 ± 196	731 ± 140	0.685	0.112	0.041
Trail Making Test part B, s	108 ± 52	103 ± 54	117 ± 58	98 ± 50	0.811	0.860	0.591
Hand reaction time, ms	260 ± 36	276 ± 49	242 ± 41	238 ± 42	0.239	0.004	0.505
Attention Network Test - conflict, ms	152 ± 63	114 ± 57	127 ± 82	117 ± 53	0.394	0.088	0.044
Patient Health Questionnaire – 9, score	3.4 ± 3.0	2.8 ± 2.8	3.0 ± 3.4	2.4 ± 3.0	0.945	0.434	0.213
Iconographical – Falls Efficacy Scale, score	13.8 ± 4.3	10.2 ± 7.0	16.3 ± 5.3	13.3 ± 8.9	0.173	0.775	0.854

Higher scores are better for European Quality of Life – 5 Dimensions, sensor-based full tandem stance and knee extension strength (for all other scores lower values indicate performance improvement)

^a^Comparing high-adherers and control group

Additional subgroup analyses based on baseline fall risk (cut-point = 0.540, median split for PPA score at baseline) showed a significant three-way interaction between high fall risk (PPA >0.540), low fall risk (PPA <0.540) and the control group (F_2,128_ = 6.90, *p* = 0.001). Two-way, post hoc analyses revealed that the high-fall risk group had a significant larger decrease in PPA score compared to the low-fall risk (*p* = 0.003) and control group (*p* = 0.001). Total exercise duration was higher in the high-risk group (15.8 h, SD 22.9) than in the low-risk group (9.4 h, SD 14.7); however, this difference was not statistically significant.

## Discussion

Our study findings suggest the ICT-based iStoppFalls exercise program is feasible for use in the homes of older community-dwelling people with initial instructor support. There were no adverse events or falls reported related to undertaking the intervention, suggesting that the unsupervised, home-based exercise program using the iStoppFalls system is feasible in terms of safety for older people. Overall, the exercise program was enjoyed by the participants and its usability was acceptable, however more work is needed to optimize adherence to the program. In line with our hypotheses, there was a larger reduction in our primary outcome of physiological fall risk in the intervention group compared to the control group, and also a small improvement in dual-tasking ability while walking. The intention-to-treat (ITT) analyses did not show a significant effect on the second primary outcome quality of life. Participants who exercised for more than 90 min per week (high-adherers) reduced their physiological fall risk significantly more than the control group. In addition, the sway area, stepping reaction time and ANT conflict measurements decreased, full tandem stance time increased and there was a trend for improved quality of life in the high-adherers.

The iStoppFalls exercise program reduced physiological fall risk in the study sample. The significant reduction in overall physiological fall risk scores (33.9 % ITT, 53.6 % high-adherers) was due to small cumulative improvements in some of the PPA sub-components (i.e., visual contrast sensitivity, quadriceps strength and postural sway). This pattern of small, cumulative benefits across physiological domains following an exercise intervention has also been reported in a previous study targeting older people who had recently been discharged from hospital [40]. However, while the overall reduction in PPA score seems clinically meaningful, it needs to be demonstrated in further studies that the program is equally effective in improving the core outcome measures of balance and muscle strength in a larger population of older people to warrant recommendation as a fall prevention intervention. Furthermore, the significant difference in reaction time is largely due to an unexpected worsening in the control group, instead of an improvement in the intervention group. On the other hand, our subgroup analyses showed that participants with higher physiological fall risk benefited most from the intervention, suggesting that the iStoppFalls system might be particularly useful in people at increased risk of falling.

Dual-tasking ability has been consistently associated with falls, and improving this ability through task-specific training might benefit people during daily life activities and possibly prevent falls. Similar to other studies including interactive exergaming [39, 41], our system was able to improve the motor component of dual–tasking ability as participants in the intervention group improved their 10-m walk times while counting backwards. In higher exergame levels, dual task components (e.g., memorising an object and identifying it from a selection at a later stage) were added to provide additional cognitive stimuli. However, it should be noted that the improvement was relatively small, but statistically significant. In relation to more specific cognitive measures, no intervention effects were evident. There was an indication that executive function could be improved in participants who exercised 90 min or more per week. One possible explanation could be that the dual task training as part of the higher exergame levels was not challenging enough for the included participants to improve other aspects of cognitive function.

Previous studies have found that increasing physical activity is effective in enhancing quality of life of older people over relatively short periods of time [42, 43]. In the current study, there was no significant improvement in quality of life following the intervention. However, there was a trend indicating improved quality of life in the intervention group participants who exercised for 90 min or more. Therefore, it will be important for older people to incorporate exercise as delivered through the iStoppFalls system into their lifestyle at higher doses in order to improve their quality of life [44].

Older people often use stepping as a strategy to regain postural stability after balance is lost in order to prevent a fall [45]. Impairment in stepping reaction time has been reported previously to be an independent predictor of falls [46]. In the present study, high-adherers (>90 min exercise per week) significantly improved their stepping reaction time compared to the control group. The ability to step faster may help older individuals to withstand unexpected balance perturbations and therefore reduce the risk of falling.

Strengths of the iStoppFalls system include the automatically-generated exercise adherence data for frequency, duration and intensity/game level, automatic reminders to exercise, and the provision of in-home instructions, immediate performance feedback and scoring information. Additionally, it has been shown that the fall risk assessment function of the iStoppFalls system was feasible for regular self-assessment of fall risk at home and that it was able to discriminate well between older fallers and non-fallers [47]. Another advantage of the Kinect-based system is that no further physical equipment (e.g., balance board) is needed to perform the exercises compared to other exergame systems.

The study had several limitations. With respect to the dose–response relationship, the low adherence rate may explain the limited improvement of secondary outcome measures. The cut-off point based on weekly exercise of 90 min duration was well below the recommended duration of 180 min per week. In this context it has to be considered that we were able to measure the “real” exercise time (i.e., core exercise excluding breaks or instructions – usually included in most intervention studies) very accurately with the iStoppFalls system. However, the ambitious goal which was based on recommendations for fall prevention exercise may have discouraged some participants [15]. A meta-analysis on adherence to home exercise programs to prevent falls by Simek et al. [48] reported 21 % fully adherent participants (95 % confidence interval: 15–29 %, range: 0-68 %). Our study reported that 23 % of all intervention participants adhered to a training dose of 90 min per week (which is similar to the most commonly prescribed dose across home-based exercise trials [48]). In this context, it is noteworthy that the power calculation was based on participants exercising more frequently, and the low exercise stimulus may explain the lack of statistically significant effects on a wide range of secondary outcomes. On the other hand, based on the results from our pre-planned subgroup analyses, we can suggest that half the recommended training dose was sufficient to induce some positive training effects.

A recent systematic review concluded that virtual reality gaming systems can be used by older people at home, but feasibility was generally poorly described [49]. The authors also reported that standby technology assistance, close monitoring and home visits were required in many studies. In our study, at least one additional house visit per participant in combination with technical support by telephone and PC remote had to be applied. It is possible that these technical difficulties may have prevented regular exercising (especially in the first month of the trial), which could further explain the low adherence rates. Therefore, more research and development work is needed before this approach can be recommended as an unsupervised training program.

## Conclusions

In conclusion, the 16-week iStoppFalls exercise program reduced physiological fall risk as hypothesized and additional subgroup analyses revealed that intervention participants with better adherence also improved in postural sway, stepping reaction and executive function. Our study findings suggest that iStoppFalls is feasible for use in the homes of older community-dwelling people with initial instructor support. The relatively low adherence to the recommended dose, which can be largely explained by the pioneering use of new technology, indicates that further research and development is required to improve the adherence and thus the effectiveness of exergame systems for fall prevention.

### Informed consent

All procedures followed were in accordance with the ethical standards of the responsible committee on human experimentation (institutional and national) and with the Helsinki Declaration of 1975, as revised in 2000 (5). Informed consent was obtained from all patients before being included in the study.
